# Feasibility of exoscopic keyhole surgery: case series

**DOI:** 10.3171/2023.10.FOCVID23116

**Published:** 2024-01-01

**Authors:** Miguel Sáez-Alegre, Christian Ríos-Vicil, Keaton Piper, Edinson Najera, Walter C. Jean

**Affiliations:** 1 Division of Neurosurgery, Lehigh Valley Fleming Neuroscience Institute, Allentown, Pennsylvania;; 2 Department of Neurologic Surgery, Mayo Clinic, Rochester, Minnesota;; 3 Department of Surgery, Section of Neurosurgery, University of Puerto Rico, San Juan, Puerto Rico; and; 4 Department of Neurosurgery & Brain Repair, Morsani College of Medicine, University of South Florida, Tampa, Florida

**Keywords:** keyhole, exoscope, illumination, neurosurgery

## Abstract

Keyhole approaches, performed with the endoscope, microscope, or exoscope, aim to minimize tissue traumatization while maximizing surgical view. The exoscope can provide better ergonomics than the microscope without restricting the space inside of the keyhole, as when using the endoscope. However, a frequently quoted reason for intraoperative exoscope-to-microscope conversion is the absence of sufficient light. In this video, the authors present 4 patients who underwent posterior fossa keyhole surgery without intraoperative conversion. The surgical objective was achieved in all patients without associated morbidity. After adequate adaptation, the exoscope allows sufficient light in the surgical field to perform safe keyhole surgery.

The video can be found here: https://stream.cadmore.media/r10.3171/2023.10.FOCVID23116

## Transcript

In this video, we present our experience with exoscopic keyhole surgery. With a series of cases, we will try to demonstrate that the exoscope provides enough light to perform keyhole surgery and discuss its benefits compared to the operative microscope and endoscope.

### 0:35

Developed by Perneczky, among others, keyhole approaches aim to maintain sufficient surgical exposure while minimizing trauma to tissue, including skin, muscle, skull, and brain.^1^ To visualize an operation through a keyhole, surgeons rely on both the microscope and endoscope. The microscope has a greater light-to-target distance, and the small size of the keyhole may limit the amount of light reaching the surgical target. This phenomenon impacts multiple aspects of the operation, including surgeon ergonomics as the operating surgeon tries to improve lighting by tilting the microscope through various angles.

### 1:14

For the endoscope, the light source is placed beyond the keyhole and much closer to target, and this provides a large amount of light even in deep fields. The cost is space. Since the endoscope itself is within the surgical corridor, the space that it takes up often restricts the surgical freedom in the keyhole.^2,3^

### 1:34

What about the exoscope? Compared to the microscope, the exoscope has a smaller light-to-target distance. Reducing this distance can, in theory, increase the amount of light that gets through the keyhole. Another benefit is that the entire surgical team shares the same view and in three dimensions.^2,3^

### 1:54

In preliminary laboratory testing, the 3D exoscope with its duo light cone seemed capable of bringing sufficient lighting through small keyholes. We will demonstrate the feasibility of exoscopic keyhole surgery through four examples. All four involve the posterior fossa, where patient positioning added another layer of challenge for visualization and illumination.

## Cases

### 2:13

Our first case is a 58-year-old male with a remote history of nephrectomy for renal cell cancer. He presented to our institution with headaches, altered mental status, and weight loss for 2 months. Further imaging discovered diffuse metastatic disease and a solitary mass in the pineal region. After a failed ETV for hydrocephalus, our tumor board recommended tumor resection for symptom control and tissue diagnosis.

Multiple methods to measure the tentorial angle have been described. We favor the one published by our senior author: in this case, 45.6°. We consider this tentorium steepness favorable for the SCIT approach.

### 2:53

The patient was taken to the OR and a right-sided paramedian SCIT approach was performed.

### 3:00

Here we have two photos of our setup in the OR. Compared to the microscope, a key difference in surgeon ergonomics is noted. With the exoscope, our hands lie below the level of our shoulder, while with the microscope, they are above this level. This latter setup leads rapidly to fatigue.

### 3:19

After performing the paramedian craniotomy, the dura was opened, and the cerebellum was mobilized inferiorly assisted by gravity. The thick arachnoid covering the pineal region was incised. Tumor was then resected using microsurgical technique. In this case, the endoscope was used to look for residual tumor. Of note, these tools should not be mutually exclusive and must be used to complement each other. The rest of the cases, however, were done only with the exoscope. The dimensions of our craniotomy were about 2 by 3 cm. Immediate postoperative MRI shows gross-total resection of the tumor. The patient woke up with no new neurological deficits; however, underwent severe respiratory failure in the postoperative period and after discussion with the family was transitioned to palliative care.

### 4:11

The next case is a 70-year-old female with 3-year history of right-sided progressive hearing loss and tinnitus and right V2/V3 numbness. Her audiogram showed right moderate to severe sensorineural hearing loss. The MRI showed a lesion centered in the CPA most likely representing a Koos IV vestibular schwannoma with no hydrocephalus. Patient still had serviceable hearing on the right. So, a right retrosigmoid approach for brainstem decompression and intended subtotal resection was planned.

This is our setup in the OR. The patient was placed in a lateral decubitus position and a C-shaped retrosigmoid incision was planned.

### 4:47

After the craniotomy, the dural opening, and CSF evacuation, the tumor was encountered. Cerebellum was mobilized posteriorly to allow access to the CPA. After confirmatory VII nerve stimulation, the capsule was incised and the tumor was internally debulked. Capsule then was detached from the brainstem. The tumor was found to be adherent to the brainstem and residual was left by intention. Postoperative, her right V2/V3 numbness was gone and hearing remained stable with HB grade I bilaterally. A watch-and-wait attitude was adapted for the residual tumor with no progression 1 year postoperative.

### 5:26

The next case is a 65-year-old female with a V2/V3 right trigeminal neuralgia refractory both to GKS and medication. The FIESTA MRI sequence showed a loop of the SCA touching the right trigeminal nerve. An MVD through a retrosigmoid craniotomy was performed. In the video, we can see the trigeminal nerve completely thinned, probably due to chronic compression plus GKS. Carefully, the nerve was separated from the SCA loop and a Teflon was placed between these two structures. Patient remains pain free 1 year postoperative on medication.

### 6:04

The last patient is a 60-year-old female with a left petrous meningioma that showed radiographic progression. The patient was asymptomatic. A left retrosigmoid approach was chosen. Her MRI showed this petrous lesion with dural tail representing a probable petrous meningioma. Again, after the craniotomy, dural opening, and CSF evacuation, the tumor is encountered. Copious venous bleeding from the tumor was found initially during the resection. The tumor was debulked and detached from surrounding structures. After initial debulking, the deep dissection started caudally from the IX-X-XI complex at its entry in the jugular foramen. VII nerve is seen and identified anteriorly to the VIII nerve and the labyrinth artery. VI cranial nerve is seen deep in the field entering the Dorello’s canal. Note that there is enough light to see this deep structure. Lastly, the 2.5-cm-diameter size of the craniotomy can be appreciated. Immediate postoperative image showed GTR of the lesion.

## Discussion

### 7:10

Compared with the endoscope, the exoscope has a greater light-to-target distance, but as our cases showed, the exoscope brings plenty of light through small keyholes. Whereas the lateral or sitting position can be challenging for the microscope, the exoscope provided great visualization and illumination without ergonomic obstacles for the surgeon.

### 7:30

However, the use of an exoscope does involve a significant learning curve. Its position relative to the surgeon, its manipulation, and even the placement of the 3D screens—these may not be intuitive to all surgeons. In addition, some users and viewers may get motion sick with the exoscope.^2,3^

### 7:48

A recent review study showed that when used in cranial and spinal cases, the exoscope had a similar complication rate compared with the operative microscope.^4^ The main reasons to switch from the exoscope to the microscope were poor illumination and 5-ALA use.^5^ More recently, the exoscopes also now include 5-ALA fluorescence. As for the illumination, our recorded data showed that the exoscope provides enough lighting even through small keyholes.

### 8:15

Analyzing the study with the highest switch rate, we found that 90% of the surgeons switched to the microscope in the first period of the study, and this dropped to 52.5% in the second half. In this study, the main reason was the learning curve.^6^ The exoscope, again, is just another tool and, as such, should be used for the benefit of our patients. Its use requires a learning process, and ease of use which comes with familiarity.

## Conclusions

### 8:41

The endoscope is a safe alternative to the microscope for keyhole surgery. Compared to the microscope, our experience showed that the exoscope has better ergonomics, is easily manipulated, and engages the whole team better.

Most importantly, it also brings in enough light to perform safe keyhole surgery.

## Disclosures

Dr. Sáez-Alegre reported being the CEO of Levendis Ltd. Dr. Jean reported consulting for Stryker and Surgical Theater outside the submitted work.

## Author Contributions

Primary surgeon: Jean. Assistant surgeon: Sáez-Alegre, Piper, Najera. Editing and drafting the video and abstract: all authors. Critically revising the work: all authors. Reviewed submitted version of the work: all authors. Approved the final version of the work on behalf of all authors: Sáez-Alegre. Supervision: Jean.

## Supplemental Information

### Previous Presentations

Portions of this video were presented at the North American Skull Base Society (NASBS) 32nd Annual Meeting held in Tampa, Florida, February 17–19, 2023.

### Patient Informed Consent

The necessary patient informed consent was obtained in this study.
